# Alterations in Molecular Profiles Affecting Glioblastoma Resistance to Radiochemotherapy: Where Does the Good Go?

**DOI:** 10.3390/cancers14102416

**Published:** 2022-05-13

**Authors:** Juliana B. Vilar, Markus Christmann, Maja T. Tomicic

**Affiliations:** Department of Toxicology, University Medical Center, Obere Zahlbacher Str. 67, D-55131 Mainz, Germany; jbrandst@uni-mainz.de

**Keywords:** radiotherapy, temozolomide, acquired resistance, mutational background, apoptosis, senescence, stemness, autophagy, cellular homeostasis alterations, malignant glioma

## Abstract

**Simple Summary:**

Glioblastoma is a type of brain cancer that remains incurable. Despite multiple past and ongoing preclinical studies and clinical trials, involving adjuvants to the conventional therapy and based on molecular targeting, no relevant benefit for patients’ survival has been achieved so far. The current first-line treatment regimen is based on ionizing radiation and the monoalkylating compound, temozolomide, and has been administered for more than 15 years. Glioblastoma is extremely resistant to most agents due to a mutational background that elicits quick response to insults and adapts to microenvironmental and metabolic changes. Here, we present the most recent evidence concerning the molecular features and their alterations governing pathways involved in GBM response to the standard radio-chemotherapy and discuss how they collaborate with acquired GBM’s resistance.

**Abstract:**

*Glioblastoma multiforme* (GBM) is a brain tumor characterized by high heterogeneity, diffuse infiltration, aggressiveness, and formation of recurrences. Patients with this kind of tumor suffer from cognitive, emotional, and behavioral problems, beyond exhibiting dismal survival rates. Current treatment comprises surgery, radiotherapy, and chemotherapy with the methylating agent, temozolomide (TMZ). GBMs harbor intrinsic mutations involving major pathways that elicit the cells to evade cell death, adapt to the genotoxic stress, and regrow. Ionizing radiation and TMZ induce, for the most part, DNA damage repair, autophagy, stemness, and senescence, whereas only a small fraction of GBM cells undergoes treatment-induced apoptosis. Particularly upon TMZ exposure, most of the GBM cells undergo cellular senescence. Increased DNA repair attenuates the agent-induced cytotoxicity; autophagy functions as a pro-survival mechanism, protecting the cells from damage and facilitating the cells to have energy to grow. Stemness grants the cells capacity to repopulate the tumor, and senescence triggers an inflammatory microenvironment favorable to transformation. Here, we highlight this mutational background and its interference with the response to the standard radiochemotherapy. We discuss the most relevant and recent evidence obtained from the studies revealing the molecular mechanisms that lead these cells to be resistant and indicate some future perspectives on combating this incurable tumor.

## 1. Introduction

Primary tumors of the central nervous system (CNS) are, in respect to 5-year survival, among the top three most lethal cancers, only behind mesothelioma and pancreatic cancer [1,2,3]. However, since brain/spinal cord tumors comprise different entities, patients having the most aggressive form, *glioblastoma multiforme* (GBM, CNS WHO grade 4), survive an average 12–15 months from diagnosis [4], with a 5-year survival rate of 7.2%, surpassing the lethality of any other type of cancer [3]. Although gliomas have a low annual incidence (6 in 100,000 cases), currently it is increasing 3% per year [5]. Particularly if unresectable, many patients diagnosed with this type of cancer suffer from impairment in neurocognition, leading to behavioral, emotional, and other cognitive troubles, which often deteriorates with time. Long-term survivals continue to have significant symptom burden and care needs [6,7].

In 2021, the new WHO classification of CNS tumors was published [8,9]. In comparison to the WHO classification of 2016, this one relies on additional molecular markers introducing more accuracy and refinement in the definition of different classes of CNS tumors, including glioblastomas (GBM, CNS WHO grade 4). The 2021 classification no longer differentiates between primary and secondary GBMs. Particularly in respect to GBMs, it has a high clinical impact, affecting histologically lower astrocytoma grades that according to a specific molecular signature can be re-classified to GBMs. However, since there are not yet statistics in respect to correlation between molecular profiles, acquired therapy resistance, and corresponding clinical outcomes, as based on this new classification, information is rare, we have predominantly concentrated on the molecular profiles, pathways, and clinical outcomes based on the 2016 WHO classification [10], but take into consideration the data with primary GBMs that correspond to the new classified ‘GBM, *IDH*-wildtype’ (CNS WHO grade 4) tumor entity.

The last significant advance in GBM treatment was acquired more than 15 years ago with the implementation of the Stupp protocol [11]. At the time, the standard therapy was maximal surgical resection followed by radiotherapy (RT), and numerous studies demonstrated no survival benefit of adjuvant drugs such as nitrosoureas compared with radiotherapy alone [11]. The Stupp protocol established the methylating agent, temozolomide (TMZ), as the adjuvant chemotherapeutic drug. In essence, the regimen remained the same over the years. It consists of the fractionated focal irradiation in daily fractions (2 Gy given 5 days per week for 6 weeks, for a total of 60 Gy) and concomitant daily TMZ (75 mg per square meter of body-surface area per day, 7 days per week from the first to the last day of RT), followed by six cycles of adjuvant high-dose TMZ (150 to 200 mg per square meter for 5 days during each 28-day cycle). The addition of TMZ resulted in clinically significant prolongation of 2.5 months in overall survival rates and an increase of 16.3% in two-year survival rates [4].

Nevertheless, tumor cells that escape the standard treatment acquire resistance and regrow. The recurrence of high-grade gliomas under/after the radiochemotherapy (RCT) is currently inevitable and poses the main obstacle for improvement in the clinical outcome. Reported results from multiple chemotherapy options so far are largely discouraging in terms of overall survival and quality of life of the patients [12,13,14]. As a consequence, no standard second-line treatment has been determined. Upon recurrences, re-challenge with TMZ is an option, and other agents such as carboplatin, etoposide, irinotecan, and nitrosoureas (ACNU—nimustine, BCNU—carmustine, CCNU—lomustine) can be employed alone or in combination, also with TMZ [15]. In this context, it is of great importance to understand the mechanisms involved in tumor cellular response to RT and TMZ.

RT, also known as radiation therapy, is a treatment modality based on the use of high-energy rays or radioactive substances to damage tumor cells with the aim to induce death and/or arrest tumor growth. It is estimated that 50% of cancer patients will receive RT, making it an important tool in the treatment of a variety of cancers [16]. The type of radiation used in RT is the ionizing radiation because it forms ions in the cells of tissues it passes through. The higher the energy of the radiation, the deeper it can penetrate the tissues. For brain tumors, usually X-rays or gamma-rays are administered [17]. In the 1960s, the concept of radiation fractionation was introduced: a treatment scheme in which the radiation dose is split and given at different time intervals to minimize normal tissue toxicity and kill tumor cells [18]. The effects of ionizing radiation on cells are well known and are divided into direct and indirect effects. The direct effects are the result of the collision of the highly energetic particle or wave with the DNA, leading to single and/or double-strand breaks (SSBs and DSBs, respectively). The indirect effects are the consequences of the reaction of the ionizing particle with other molecules present in the cell, such as water, proteins, and lipids [16,19,20]. It is known, however, that most of the radiation-induced lesions are a consequence of the reactive oxygen species (ROS) generated by the radiolysis of the water, due to the abundance of this molecule in the cell. This oxidized state of the cell also strongly induces inflammation [21,22].

**Figure 1:**
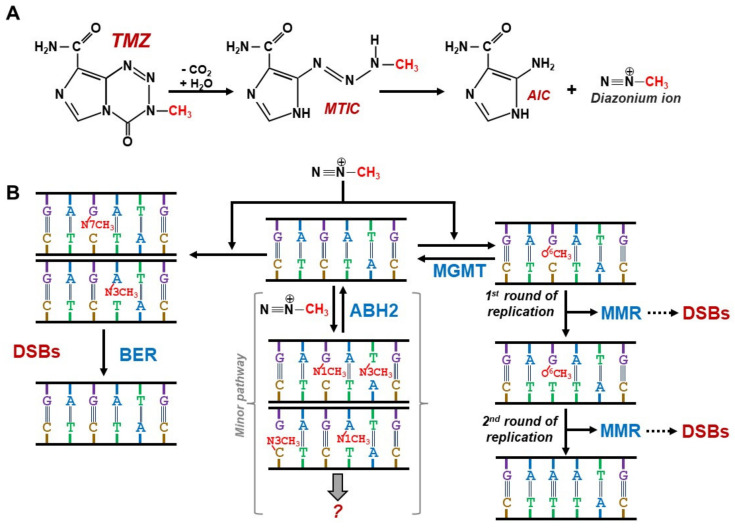
Metabolization pathway of temozolomide (TMZ) (**A**) and the consequences of the main mutagenic lesion (O^6^mG) and N—alkylations on DNA, depending on the DNA repair mechanisms induced (**B**). For a full description, see the main text.

TMZ is an acid-stable prodrug, allowing for 100% oral availability and rapid absorption. Its lipophilic nature enables it to cross the blood–brain barrier (BBB). Under neutral or alkaline conditions, it undergoes hydrolytic ring opening, giving rise to the first significant intermediate—MTIC (5-(3-methyltriazen-1-yl) imidazole-4-carboxamide). This dissociates to AIC (4-amino-5-imidazole-carboxamide) and liberates methyl diazonium ions, which then react with nucleophilic sites on DNA [23] (Figure 1A). Its cytotoxicity is mediated by the addition of methyl groups on N7 and O^6^ sites on guanines (70% N7mG and 6% O^6^mG of the lesions, respectively) and N3 sites on adenines (10% N3mA of the lesions) [24,25,26].

Although more abundant, the N7mG and N3mA lesions do not cause any mismatches and can be quickly repaired by the base excision repair (BER), while the N1mA and N3mC can be directly reversed by the human protein ALKB homolog 2 (ALKBH2) [24,27] (Figure 1B). The O^6^mG, on the other hand, can have two outcomes: briefly, in the presence of the suicide protein MGMT (O^6^-methylguanine DNA-methyltransferase), O^6^mG is repaired after the methyl group is recognized and transferred to the enzyme’s cystein residues (direct repair = DR or direct reversal) (Figure 1B). In the absence of MGMT, O^6^mG mispairs with thymine (T) and this mismatch is recognized by the mismatch repair (MMR) pathway. However, the T in the newly synthesized strand is recognized as the wrong base and therefore removed. Because the O^6^mG lesion remains in the DNA, it leads to repeated incorporation of T opposite to O^6^mG by the polymerase and MMR recruitment. These futile cycles of the thymine incorporation, MMR activation and thymine removal lead to dTTP depletion and DNA strand breaks [28,29] (Figure 1B).

Thus, RT and TMZ treatments convey to the same kind of major lesions—DNA SSBs and DSBs. It is common to assume that given enough lesions, the cells will no longer be able to cope with damage and die. How the cells really respond to these lesions is quite complex. Different players take place to induce cell cycle arrest, DNA repair, autophagy, senescence, stemness, and/or apoptosis. In this review, we will discuss the main pathways and alterations in cellular homeostasis described to influence the GBM cellular outcome towards radiation and/or TMZ treatment, underlining the mechanisms leading to acquired resistance.

## 2. The Landscape of Genetic and Genomic Alterations in GBMs

The last decades of research have resulted in the identification of various chromosomes and genes that are frequently altered in GBMs. Considering the 2016 WHO classification, molecular genetic analyses have shown a different pattern of alterations in primary (90% of the GBM cases), secondary (10% of the cases), and treated relapsed tumors, [10]. The first well-established alterations were relative to isocitrate dehydrogenase *IDH1*/*2* mutations, which became a factor for categorization and prognostication of these tumors [30]. The enzymes encoded by this group of genes work on chromosome remodeling/DNA methylation and metabolic signaling. Historically seen, mutated *IDH1*/*2* allowed for clear differentiation between primary and secondary GBMs: it was frequent in secondary tumors and quite rare in primary or infant ones [31,32]. However, as already mentioned, the new 2021 WHO CNS classification does not differentiate between primary and secondary GBMs, and there is no entity such as ‘GBM, *IDH*-mutant’ [9,33]. Strictly speaking, the new classification defines GBM as ‘glioblastoma, *IDH*-wildtype’ (CNS WHO grade 4), with either one of the following altered molecular diagnostic markers [34]: *TERT* promoter mutation (~80%), simultaneous gain of chromosome 7 and loss of heterozygosity of chromosome 10q (+7/—0) [35], amplification and rearrangement of receptor tyrosine kinases, most commonly affecting *EGFR* (~50%) [34,36]. Thus, the new classified GBM, CNS WHO grade 4 entity reflects the primary GBM (WHO grade IV, according to the 2016 WHO classification), and overexpresses wild-type IDH1/2 protein [37]. The *IDH1* gene mutations are often substitutions within exon 4 in the codon 132 and correspond to a more favorable outcome for the patient [38]. Likewise, there are also other quite rare but favorable *IDH1/2* mutations [39]. According to the 2021 classification, they describe a new classified ‘astrocytoma, *IDH*-mutant’ tumor entity [8,9].

**Figure 2:**
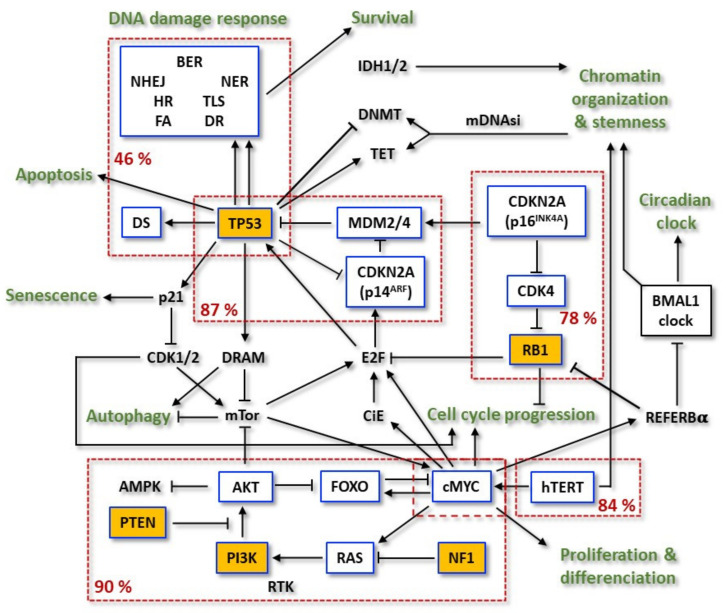
Flowchart of the most frequent mutations in the relevant pathways involved in primary GBM resistance. Within the boxes outlined in blue are significantly mutated genes (SMG) and in the yellow boxes are the driver-mutated genes. Dashed red boxes group mutated genes of the same pathway and display the relative mutation frequency of the pathway. Adapted from TCGA.

It is important to underline that the new 2021 classification is a further evolvement of molecular profiling, combined with histologic tools, in diagnosis of the CNS tumors that was for the first time introduced by the breakthrough 2016 WHO classification, which integrated molecular and histologic features to refine classification of those tumors. Thus, other alterations in primary GBMs were recognized comprising the tumor suppressor genes (TSGs) *TP53*, *RB1*, *CDKN2A (p16^INKA4^/p14^ARF^)*, and *PTEN* and the proto-oncogenes (POGs) *EGFR*, *PDGFR*, *MET*, *CDK4*, *CDK6*, *CCND1*, *CCDN3*, *MDM2*, *MDM4*, and *MYCC* [40] (Figure 2).

Different from the most TSGs, *TP53* is rarely deleted in GBM [41]. The majority of its mutations are missense mutations and occur in the DNA binding domain (DBD), which leads to the inhibition of its transcriptional activity. The corresponding mutated protein is constitutively overexpressed and takes over oncogenic functions, not present in the wild-type (wt) form. These p53 mutants (mt) are called gain-of-function (GOF) p53mt [41,42,43]. On the other hand, mutations at other sites can bring out radically different cellular effects.

Further and more detailed discoveries were obtained with The Cancer Genome Atlas (TCGA) project, which aimed to catalogue cancer-causing genome alterations in large cohorts of human tumors using multi-dimensional analysis. GBM was the first cancer systematically studied by TCGA [40]. The first TCGA work analyzed 206 GBMs and, in addition to the previously described alterations, detected new significantly recurrent deletions involving *NF1* and *PARK2*, and amplification of *AKT3*. PI3K complex activating missense mutations were also frequent. Rare, but informative events were also computed, such as *FGFR2* and *IRS2* amplifications and *PTPRD* deletion. Chromosome 7 gain (+7) (associated with *EGFR* amplification) and loss of heterozygosity of chromosome 10q (—10) (associated with *PTEN* loss) were among the most frequent genetic alterations [34,44,45,46]. For single nucleotide polymorphisms (SNPs) detection, 601 selected genes in 91 matched tumor-normal pairs (72 untreated and 19 treated cases) were sequenced. The results uncovered 453 validated non-silent somatic mutations in 223 unique genes. The background mutation was four times higher in treated tumors. Hypermutator phenotypes were described in 7 of the 19 samples treated with TMZ and/or CCNU (lomustine) and were associated with mutations in at least one MMR gene; 48.5% of the tumors exhibited *MGMT* promoter methylation [36].

Later, TCGA expanded the analysis to more than 500 primary GBM samples [36]. Due to the nature of these tumors, *IDH1* mutations were infrequent. Of those, 291 paired, tumor-normal genomic DNA samples were subjected to whole-exome sequencing (WES). Among the somatic mutations, 20,448 single nucleotide variants (SNVs), 39 dinucleotide mutations and 1153 small insertions and deletions (indels) were detected. Investigation of significantly mutated genes (SMGs) identified *PTEN*, *TP53*, *EGFR*, *PIK3CA*, *PIK3R1*, *NF1*, *RB1*, *IDH1*, *PDGFRA*, and the newly described *LZTR1* as driver-mutated genes in GBMs (Figure 2). Interestingly, the protein encoded by *LZTR1* is exclusively located at the Golgy network and, therefore, it is suspect to stabilize this complex. Other genes were also shown to have frequency above background, such as *SPAT1*, *ATRX*, *GABRA6*, and *KEL*. In the expanded analysis, the most common amplification events on chromosome 7 (*EGFR*/*MET*/*CDK6*), chromosome 12 (*CDK4* and *MDM2*), and chromosome 4 (*PDGFRA*) were found to be in higher frequencies than previously reported. Single gene deletion targets included *LRP1B*, *NPAS3*, *LSAMP*, and *SMYD3* [36,40].

Overall, 46% of the samples from a study were found to harbor at least one nonsynonymous mutation in a set of 161 genes functionally linked to chromatin organization. Telomerase reverse transcriptase (*TERT*) activating mutations and its subsequent higher mRNA expression were observed. Two hot spot mutations in the *hTERT* promoter region were mutually exclusive between each other and reached 84% of the cases [47]. On the other hand, *hTERT* mutations were also found to be mutually exclusive with *ATRX* mutations, concurrent with *IDH1* and *TP53*. Receptor tyrosine kinases (RTKs) were found altered in 67.3% of the tumors: *EGFR*—57.4% (mutant variant vIII as the most common), *PDGFRA*—13.1%, *MET*—1.6% and *FGFR2/3*—3.2%. *PI3K* mutations were found in 25.1% of GBMs. Considering *PI3K* and *PTEN*, 89.6% had at least one alteration in the PI3K pathway, and 39% had two or more. TSG *NF1*, a GTPase-activating protein that negatively regulates the RAS/MAPK pathway, was deleted or mutated in 10% of cases and never co-occurred with *BRAF* mutations (2%). The p53 signaling pathway was found to be dysregulated in 85.3% of tumors, through mutation/deletion of *TP53* (27.9%), amplification of *MDM1*/*2*/*4* (15.1%), and/or deletion of *CDKN2A* (57.8%). The RB pathway was also disturbed by different events: 7.6% by direct *RB1* mutation/deletion, 15.5% by amplification of *CDK4*/*6*, and the remainder via *CDKN2A* deletion [47,48,49].

Gene fusions and translocations in GBM have also been subject of investigation since they have the potential to create chimeric proteins with altered functions. Additionally, they can rearrange gene promoters that can lead to increased oncogene overexpression and activity or decreased expression of TSGs. These events can be very specific for the cancer type and can constitute a diagnostic marker. As an example, it is known that the translocation of the Philadelphia chromosome 9–22, which results in the *BCR-ABL1* gene fusion, is characteristic of the chronic myeloid leukemia [50]. In GBM, it was found that *FGFR3-TACC3* in-frame activating kinase fusion is a common marker and might play an important role in oncogenesis and cancer progression [51]. However, it is not quite specific to this kind of tumor.

Regarding DNA damage repair genes, it is known that these genes play key roles in maintaining genomic stability and the dysregulation of diverse repair functions is an important determinant of cancer risk, progression, and therapeutic response. Moreover, it was recently shown that DNA repair proteins may have a role beyond the level of DNA damage, revealing unexpected functions in transcriptional regulation, cancer stem cell (CSCs) signaling, and epithelial–mesenchymal transition (EMT) [52]. The prevalence of alterations in DNA repair genes was determined on 126 GBM samples. It was shown that numerous tumors harbored at least one alteration in core DNA repair genes. Five samples harbored alterations in the Fanconi Anemia (FA) pathway, three in the Non-Homologous End-Joining (NHEJ), three in Nucleotide Excision Repair (NER), one in BER, two in Translesion Synthesis (TLS), six in Homologous Recombination (HR), five in MMR, five in Damage Sensoring (DS), and 28 in DR [53]. In the assessment of the potential genomic consequences of these alterations, the authors also showed the association of TMZ signature (corresponding to COSMIC signature 11) with *MGMT* alterations [53]. The most frequent *MGMT* alteration found was the promoter methylation (92.4% of all alterations), leading to gene silencing. *EXO5* silencing (via promoter methylation) was also frequent in GBMs (46%). When broken down to subtypes, *EXO5* silencing was exclusively observed in *IDH* wild-type GBM. This observation suggests that EXO5 (5′ and 3′ bidirectional single-stranded DNA-specific exonuclease) may play a role in the pathogenesis of subsets of these cancers. For instance, recently it was shown that functional deficiency of this DNA repair gene plays a role in prostate tumorigenesis [53,54].

Another group of genes that has been implicated in cancer initiation, progression, and resistance to therapy is the group of the autophagy-related genes (ARG). Autophagy is an important homeostasis mechanism, which degrades and recycles components of the cells in lysosomes, providing materials and energy [55]. The autophagic pathway shares numerous components with the RTK/PI3K/AKT signaling. However, beyond these common players, autophagy-related proteins functionally control and regulate the autophagosome formation, including initiation, nucleation, elongation of the membrane, maturation of autophagosomes, trafficking, docking, fusion with lysosome membranes, and degradation of intra-autophagosomal contents in a hierarchical manner [56]. In this context, a significant correlation of some autophagy (ATG) gene polymorphisms and glioma susceptibility was reported [57]. Moreover, when analyzing expression data of 986 patients, a signature containing 14 ARGs able to predict GBM patients’ prognosis was obtained by researchers [58,59]. Of those, the expression of *MTMR4*, *LENG9*, *P4HB*, *PCIRG1*, *HSPA5*, *DRAM1*, *CTSD*, *S100A8*, and *CCL2* increased with signature risk score. Additionally, patients with this signature, classified as high-risk, had more malignant characteristics than those classified as low risk [58].

Cancer progression also encompasses gradual loss of a differentiated phenotype and acquisition of progenitor-like, stem-cell-like features. Undifferentiated primary tumors are more likely to metastasize and to be resistant to current therapies [60]. Malta and co-workers defined two signatures to quantify stemness based on a multi-platform analysis: one was reflective of epigenetic features (mDNAsi, DNA methylation-based stemness index) and the other of gene expression (mRNAsi, mRNA expression-based stemness index) [61]. For GBMs, they found a strong association between high pathologic grade and recently published glioma subtypes: mDNAsi was highest in highly aggressive GBMs. In addition, high mDNAsi was strongly associated with mutations in *NF1* and *EGFR* and infrequent mutations in *IDH1*, *TP53*, *CIC*, and *ATRX*. The authors also found a negative correlation between higher stemness indices and reduced leukocyte fraction and lower PD-L1 expression. These findings reveal that GBMs might be less susceptible to immune checkpoint blockade treatments due to insufficient immune cell infiltration or preexisting downregulation of the PD-L1 pathway, which makes further inhibition ineffective [61]. Indeed, large-scale phase III clinical trials with immune checkpoint inhibitors did not increase GBM patients’ survival [62,63].

Using bioinformatic tools to find common putatively actionable alterations in a set of 33 types of cancer (including GBMs), it was found that across the entire data set, *CDKN2A* deletions (13%), *PIK3CA* mutations (12%), *MYC* amplifications (8%), and *K-RAS* mutations (7%) were the most common [64,65]. Of those, *CDKN2A* loss may predict sensitivity to CDK4/6 inhibitors. The *BRAF V600R* sequence variant, which confers sensitivity to vemurafenib in melanomas, was only detected in 1.7% of the GBMs. *MYCN* mRNA expression was also found upregulated in GBMs. In an investigation on the potential differences with respect to MYC-associated pathways, GBMs were found to harbor the MYC canonical signature with respect to DNA replication/repair and chromatin. It could also be shown that they exhibit the MYCN signature associated with genes related to neuronal function and development [64,65,66]. Because the studies from the TCGA consortium have the largest sample sizes and the most comprehensive, up to date systematic analyses (from which many other studies have been out seeded), it is reasonable to assume the frequencies of those alterations as putative statistics for GBMs.

All these molecular features have led researchers to propose a taxonomic approach to GBM, dividing it into four molecular subtypes: classical, neural, pro-neural, and mesenchymal. However, the methodological analyses of the samples used for this characterization comprised not only the *bona fide* tumor cells but also the tumor-associated nonmalignant cells. A more stringent analysis, considering the separation of malignant (GBM) and nonmalignant (glial) cells led to a state-of-the-art classification into three subtypes: classical, pro-neural, and mesenchymal, suggesting the former neural subtype would arise from contamination of the samples with non-malignant cells [67,68,69,70]. Moreover, as already mentioned, recent re-classification of tumors from the CNS by the WHO 2021 [8,9,71] has taken more molecular features as diagnostic criteria into consideration. In this regard, the most relevant change refers to low-grade astrocytomas and *IDH*-mt astrocytomas. Molecular profiling showed that the overwhelming majority of histologically lower CNS WHO grade 2 and grade 3 diffuse astrocytomas exhibiting the *IDH*-wt status, shared signature in genomic alterations (either *EGFR* amplification, or concurrent +7/—10, or *TERT* promoter mutation) and clinical outcomes with the former primary GBM, WHO grade IV or currently classified GBM, CNS WHO grade 4, suggesting those tumors were only under-represented GBMs [72]. Additionally, *IDH*-mt diffuse astrocytomas are now considered entities per se (astrocytoma, *IDH*-mutant, WHO grade 2, 3, 4). These changes have recently been summarized [33].

## 3. Mechanistic Evidence of GBM Resistance to RCT

Countless studies, in vitro and in vivo, have tried to reveal the gene or the group of genes that are responsible for not only the GBM tumor initiation and growth, but, also, for the still inevitable resistance to therapy. How these genes cooperate to escape therapy-induced cell death is variable and complex, and hundreds of genes have been tested. In this review, we will focus on the analysis of the acquired GBM resistance to RCT involving major pathways correlated with the major alterations compiled above (Section 2). We are not aiming to focus on the impact of long non-coding RNAs (Lnc-RNAs), epigenetic regulators such as microRNAs (miRNAs), extracellular vesicles/microenvironment, or metabolism referring to ROS, drug efflux pumps/ABC transporters on the response of GBMs to radiochemotherapy.

### 3.1. Metabolism—Isocitrate Dehydrogenase (IDH) and the Warburg Effect

Some metabolic features are shared among all GBMs, in contrast to the normal brain. The production of lactate, together with glucose and acetate oxidation are some of them. In this context, IDH isoforms function in the catalysis of the reversible oxidative decarboxylation to yield α–ketoglutarate (α-KG) and NADPH, to provide reducing equivalents to support lipid biosynthesis and redox homeostasis in the tricarboxylic acid (TCA) cycle, also known as the citric acid cycle. Overall, 65% of NADPH in GBMs is driven by the enzymatic activity of IDH, which is reduced to 35% when it is mutated [73]. Therefore, *IDH* mutations are responsible for considerable increased oxidative burden. TMZ was found to be able to reduce expression of IDH1 in patient-derived tumor spheres [37,74]. Similarly, one study observed that targeting *IDH* expression and activity results in reduced α-KG, NADPH, deficiency in carbon flux from glucose or acetate to lipids, exhaustion of reduced glutathione, increases in ROS, and enhanced histone methylation and differentiation marker expression [75]. These findings are similar to the clinical features related to *IDH* mutations, in which the most common mutation (*IDH1*^R132H^) produces a neomorphic enzyme that converts α-KG to D-(R)-hydroxyglutarate (2-HG), leading to the accumulation of this oncometabolite, which in turn is a competitive inhibitor of α-KG-dependent enzymes (e.g., DNA and histone demethylases). These changes result in a block of cell differentiation and acquisition of a CpG island hypermethylator phenotype (CIMP) [76,77].

Under hypoxic conditions, many solid tumors, including GBM, rely on utilizing aerobic glycolysis over oxidative phosphorylation for their growth and survival, whereby it is believed that eventually MYC and HIF1 (hypoxia-inducible factor) promote this metabolic shift, known as the Warburg effect [78]. Glycolysis is a multistep process, occurring in the cytosol, in which glucose is converted into the final metabolite—pyruvate. Pyruvate enters the mitochondrial TCA cycle to serve as a substrate for acetyl-CoA. However, to promote this metabolism shift, pyruvate dehydrogenase kinase (PDK, easily confused with phosphatidine–inositol-dependent kinase 1, PDK1) is activated by diverse oncogenic tyrosine kinases localized to different mitochondrial compartments [78]. Upon tyrosine phosphorylation and activation, PDK phosphorylates and inactivates pyruvate dehydrogenase (PDH). This leads to inactivation of the PDH enzyme complex (also abbreviated as PDC) and prevents conversion of pyruvate to acetyl-CoA [79]. In the citric acid cycle in the mitochondria, acetyl-CoA is oxidized to produce energy; by downregulation/inhibition of the PDC by PDK, the oxidation of pyruvate in mitochondria is decreased and the conversion of pyruvate to lactate in the cytosol increased. GBMs often possess increased PDK levels, which contribute to resistance to the standard therapy. Therefore, reverting the Warburg effect by targeting PDK seems to be a reasonable approach for sensitization of TMZ-resistant GBM [80,81], and has been shown to play a decisive role for EGFRvIII/PDK1 axis in TMZ resistance [79].

### 3.2. DNA Damage Response (DDR) and DNA Repair

It is well established that the ultimate killing lesion of TMZ is the O^6^mG, although the other lesions might contribute to some extent, depending on the dose administered [26]. Hence, the methylation of the promoter of the MGMT protein, leading to the protein silencing (MGMT-negative tumors) was shown in one study to be the strongest predictor of survival in GBM patients [82]. However, despite the importance of the *MGMT* promoter status for the patient’s survival, i.e., the TMZ therapy outcome, the Stupp protocol also benefited patients with unmethylated *MGMT* promoter in another study, and thus it is the treatment applied in those cases [11].

The chronological molecular mechanism of action of the O^6^mG lesion has been beautifully detailed as “Although replication fork progression was unaffected in the first cell cycle after treatment, electron microscopic analysis revealed an accumulation of O^6^mG and MMR-dependent single-stranded DNA (ssDNA) gaps in newly replicated DNA. Progression into the second cell cycle required HR, while the following G2-arrest required the continued presence of O^6^mG” [83]. Therefore, the action of TMZ requires the presence of proficient MMR, which senses the primary TMZ effect—the guanine methylation. Constant activation of MMR leads to the accumulation of ssDNA, which in turn leads to the generation of dsDNA breaks (Figure 1B).

DSBs are a classical activator of ATM/ATR (ataxia telangiectasia mutated/ATM and RAD-3 related), although it is assumed that MMR itself can induce those kinases [84,85]. In MMR-proficient cells upon TMZ treatment, ATR is first activated and then ATM inducing pCHK1 (checkpoint kinase 1), pCHK2 (checkpoint kinase 2) and p53 phosphorylation at multiple sites, including Ser15 and Ser20, necessary for its stabilization and transcriptional activation and binding [84,86,87,88]. The ATM/CHK2 axis is also necessary for IR-induced response in GBMs. γH2AX, a marker for DSBs, is activated upon both treatments (TMZ and IR) but does not rely on MMR status for IR-induced response [89,90]. After TMZ treatment, MMR-deficient cells fail to activate ATM, ATR, CHK1, CHK2, p53, to arrest the cell cycle in G2/M phase and to trigger apoptosis, rendering the cells resistant to the drug. MMR components and HR players, such as MSH2, MSH6, EXO1, and RAD51, are found to be significantly downregulated after TMZ exposure [88]. In agreement with these findings, PMS2, MLH1, MSH2, and MSH6 were found to be enriched among TMZ-resistant gene candidates, using CRISPR knockout libraries [91]. Moreover, the knockdown of RAD51, BRCA1 (breast cancer 1), and the RAD51 paralog XRCC3 (X-ray repair cross complementing 3) prevented the repair of the O^6^mG-induced DSBs leading to cell sensitization, while RAD51-CRISPR activation led to TMZ resistance [91,92,93].

NHEJ seems to play different roles upon TMZ and IR treatments: whereas DNA-PKcs (DNA-dependent protein kinase catalytic subunit) inhibition only plays a minor role in TMZ resistance, it greatly sensitizes glioma cells to IR [91,92,94,95]. PAXX, a paralog of XRCC4 and of XLF, is a recently discovered factor involved in the NHEJ pathway that acts by binding with and facilitating Ku proteins accumulation at DNA ends [96]. PAXX-deficient cells showed slightly increased HR and sensitized TMZ-resistant glioma cells. However, the mechanism by which PAXX contributes to TMZ resistance is linked to its cooperation with BER polymerase beta (POLB) [97]. Little is known about NER involvement in TMZ response, but BER is thought to contribute to the removal of TMZ secondary lesions, apurinic/apyrimidinic (AP) sites and IR-induced oxidized lesions [98,99]. The BER enzyme N-methylpurine-DNA-glycosylase (MPG) is responsible for the recognition of N7mG and N3mA, the most abundant TMZ-induced lesions, and is normally expressed in GBMs [100]. Its true contribution to TMZ resistance, however, is a matter of debate. G/T mismatch-specific thymine DNA glycosylase (TDG), which in theory would be able to recognize mispaired T opposite to O^6^mG, has not been subject of investigation.

BER inhibitors, such as Methoxyamine (MeOX) and APE (apurinic/apyrimidinic endonuclease) inhibitor (APEi), have been reported to potentiate the cytotoxicity of alkylating agents through an accumulation of AP sites due to preventing AP-endonuclease dependent cleavage [101,102,103]. PARP1 (poly [ADP-ribose] polymerase 1) is a BER enzyme that deals with ssDNA to maintain genomic integrity. Its mechanism of action is by binding to DNA nicks and addition of poly ADP-ribose (PAR) to histones H1 and H2B, promoting chromatin relaxation and access to DNA repair enzymes in different pathways (BER, SSB and DSB repair) [104,105]. Most studies show a sensitization of glioma cells to TMZ after inhibition of PARP1, suggesting its implication in N7mG and N3mA removal [106,107]. PARP1 inhibition is able to restore TMZ chemosensitivity in MSH6-deficient cells, but its action has been shown to be independent of other BER components, such as APE1 and XRCC1 [108]. Taking all into account, including the systematic evidence that MGMT activity abrogates TMZ toxicity, repair of N7mG and N3mA does not seem to influence TMZ toxicity in gliomas to great extent; this is probably due to multiple coping repair mechanisms. PARP1 might not be implicated in the BER of those secondary lesions, but it might stimulate other repair proteins involved in O^6^mG processing. Indeed, several proteins of nearly all repair pathways have the PARP1 binding motif, such as BRCA1 and BRCA2 (HR), MSH6 (MMR), CETN2 (NER), NEIL3 (BER), ALKBH3 (DR), FANCG (FA), and RPA (repair accessory protein) [109]. Additional mechanisms of rescued TMZ chemosensitivity by PARP1 inhibition could be related to less affinity of repair enzymes to chromatin and to transcriptional factors alterations [56,106,107,108].

TLS is a common post-replicative DDR pathway, activated when the high-fidelity DNA polymerases (pols) encounter lesions or non-coding bases, which lead to the blockage of the replication fork and long stretches of RPA-coated ssDNA. This structure leads to RAD6–RAD18 recruitment and this complex catalyzes the addition of one moiety of ubiquitin to K164 of the resident PCNA (proliferating cell nuclear antigen). Upon the monoubiquitylation of PCNA, the replicative pols are switched for one or a set of error-prone TLS pols, to avoid the dissociation of the replisome apparatus and allow the progression of the fork [110]. After the bypass of the damage by the TLS pols, the replicative pols resume the task. RAD18 plays an important role on both IR and TMZ resistance. RAD18 has been shown to be low expressed in glioma cells and its ectopic-induced overexpression was shown to lead to an increased resistance to IR, while the knockdown rendered the cells more sensitive [111,112,113]. The knockdown of RAD18 also disrupted HR repair and provoked an accumulation of DSBs [112,114]. The ability of each TLS pol to bypass the different kinds of lesions induced by TMZ has been poorly investigated and the depletion of TLS pols by inhibitors is suggested to be an effective way to overcome TMZ resistance [115]. The nucleoside 5-nitroindolyl-2′deoxyriboside (5-NIdR) has been shown to preferentially inhibit TLS pols ƞ (eta), κ (kappa), ɩ (iota) over other pols, while indole-aminoguanidine (IAG) analogues specifically inhibit POLκ [116,117].

POLƞ is a major TLS pol, the only TLS pol which deficiency leads to cancer-related phenotype: the *Xeroderma pigmentosum* variant (XPV) syndrome [118]. XPV cells are very sensitive to IR and TMZ (Vilar, unpublished data), but the studies focusing on the significance of this enzyme in GBM context are rare [119]. POLκ, a TLS polymerase usually involved in the bypass of several types of lesions in the DNA-minor grooves, is significantly upregulated in GBM cells and tissues and correlates with a poor patient prognosis [120]. POLκ-CRISPR activation confers glioma cells’ resistance to TMZ [91]. Another study found the overexpression of this TLS pol in previously TMZ-sensitive glioma cell lines conferred the cells’ resistance, whereas its inhibition markedly sensitized TMZ-resistant cells in vitro and in xenografts. POLκ depletion also disrupted HR and impaired ATR-CHK1 signaling and cell cycle progression, via RAD17 ubiquitination and proteasomal degradation [121]. POlɩ CRISPR/CAS9 knockout glioma cells were also more sensitive to TMZ [91]. Lastly, the induction of the catalytic subunit of Polζ, named REV3L, as consequence of the disruption of the c-Myc/miR29c were shown to turn glioma cells resistant against TMZ [122]. However, REV3L is neither involved in O^6^mG tolerance nor has an influence on the BER of the N—alkylations; rather, it is likely to be involved in the tolerance of N—alkylations or AP sites originated from them, as shown with REV3L knockout mouse embryonic fibroblasts [123].

### 3.3. RTK Pathway and Wnt Signaling

**Figure 3:**
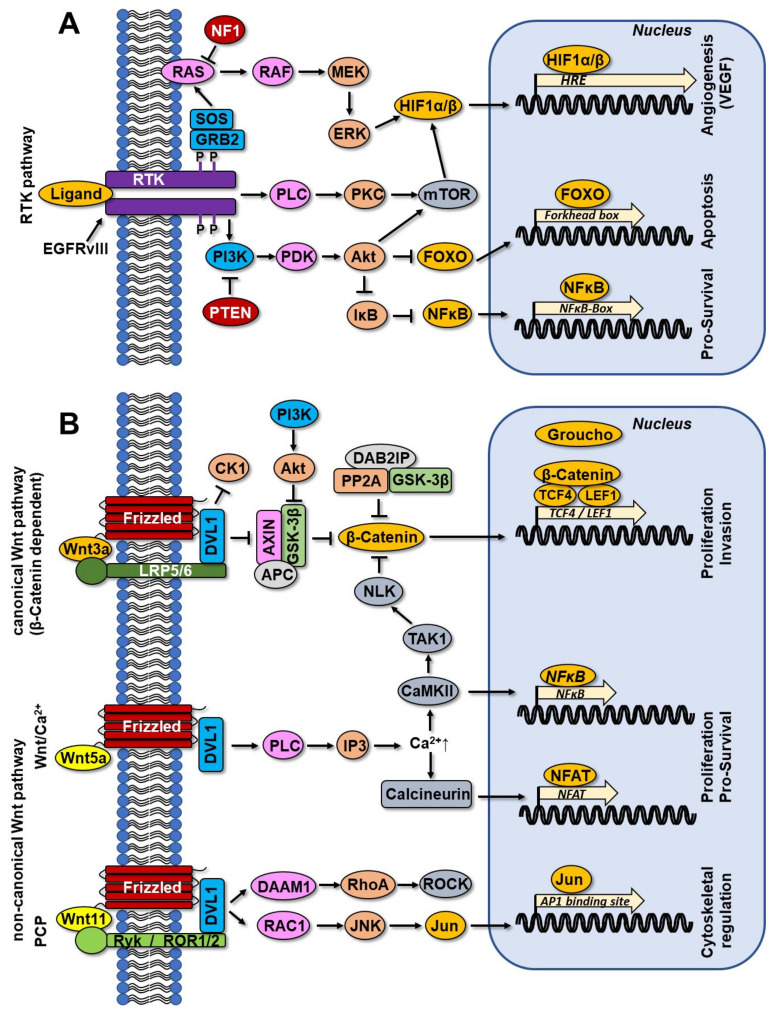
Schematic representation of the RTK pathway (**A**) and the Wnt canonical (β-catenin dependent) and non-canonical pathways (**B**). For a detailed description, see the main text.

RTKs, a family of cell surface receptors, are activated upon the binding of a corresponding ligand, resulting in receptor dimerization and autophosphorylation of the tyrosine kinase domain. Two major signaling pathways, RAS/RAF/MEK/ERK and RAS/PI3K/AKT are activated, which are responsible for tumor proliferation, invasion, survival, and angiogenesis [124] (Figure 3A). Alterations in both major pathways account for ~90% of GBM cases reported [36,40].

As already mentioned, RTKs have been found dysregulated in ~67% of GBM. The most frequent alteration found (~60%) is the amplification of the epidermal growth factor receptor (EGFR, also known as ErbB1/HER1). The truncated mutant EGFR variant III (EGFRvIII) is also frequently expressed (~50% of all GBM) and constitutively activated independent of the ligand binding [125]. Interestingly, although this EGFR variant reinforces tumor growth and migration, it seems to be a favorite prognostic marker, since overall survival of glioblastoma patients upon administration of the standard RCT is increased [126,127]. On one hand, this could be explained by the apparent neoantigen properties of EGFRvIII, eliciting immune response [124] and on the other, it has been reported that the EGFRvIII-positive and MGMT-negative (promoter-methylated) tumors upregulate MMR, thus responding better to TMZ therapy [128].

The membrane-anchored RAS is activated upon exchange of GDP with GTP, further activating mitogen-activated protein kinase (MAPK) kinase (MEK) that phosphorylates and activates the extracellular signal-regulated kinase (ERK), also known as p42MAPK [129]. The triggering of this pathway leads to activation of HIF1α, which induces transcription of the vascular epithelial growth factor (*VEGF*) as a prerequisite for angiogenesis [130]. The tumor suppressor neurofibromin (NF1) is the intracellular RAS inhibitor, which, via RAS-bound GTP hydrolysis, leads to inactivation of RAS activity [131] (Figure 3A). The *NF1* gene mutates during the process of gliomagenesis [40].

Upon activation of an RTK by a ligand, phosphatidyl inositol 3 kinase (PI3K) is translocated to the plasma membrane and is involved in converting phosphatidylinositol (4,5)-diphosphate (PIP2) into phosphatidylinositol (3,4,5)-triphosphate (PIP3). PIP3 phosphorylates and activates phosphoinositidine-dependent kinase 1 (PDK1) and AKT [132]. Phosphatase and tensin homolog (PTEN) functions as an intracellular inhibitor of PI3K signaling via dephosphorylation of PIP3 to PIP2 [133], and is found mutated or deleted in ~40% of GBM cases [134]. AKT activates the mammalian target of rapamycin, mTOR (either in mTORC1 or mTORC2 complex), that, in analogy to the MAPK/ERK pathway, directly phosphorylates and activates HIF1α, supporting proliferation and angiogenesis [135]. AKT also inhibits the members of the FOXO (forkhead box 1) subfamily, which are the activators of the diverse pro-apoptotic genes, finally leading to pro-survival effects. AKT can also directly phosphorylate BAD and GSK3, converting them into pro-survival players [132,136]. AKT can, as well, phosphorylate the inhibitor of the nuclear factor kappa-light-chain-enhancer of activated B cell (NF-κB), IκB, which undergoes proteasomal degradation, deliberating NF-κB from the inhibitory complex, which in turn translocates to the nucleus and transcriptionally activates its target anti-apoptotic genes [137].

The Wnt signaling and its contribution to oncogenesis and biology of GBM was recently summed up in an excellent review [138]. Thus, we intend to merely summarize main components along the pathway and to concentrate on the contribution of the Wnt signaling to the adapted RCT resistance. In respect to function and components’ composition, there are two basic Wnt signaling pathways: canonical and non-canonical. Canonical Wnt signaling highly depends on β-catenin, while the non-canonical pathway is independent of the β-catenin function and can be subdivided into the planar cell polarity (PCP) pathway and the Wnt/calcium (Ca^2+^)-dependent pathway (Figure 3B). The signaling along the canonical pathway initiates with the binding of a Wnt ligand to the transmembrane frizzled (FZD) receptor and to the transmembrane co-receptor, the low-density lipoprotein receptor-related protein 5 and 6 (LRP5/6). This triggers the phosphorylation of their intracellular components, enabling binding of the cytoplasmic disheveled segment polarity protein 1 (DVL1) to FZD and the axis inhibition protein (AXIN), a scaffold cytoplasmic protein, to LRP5/6. DVL1 gets activated by phosphorylation, and inhibits the activity of the glycogen synthase kinase 3β (GSK3β) and casein kinase 1 (CK1). In turn, β-catenin cannot be phosphorylated by those kinases and is not marked for ubiquitination by β-transducin repeating protein (β-TrCP). This leads to increased cytoplasmic levels of β-catenin which translocates to the nucleus and forms the transcription complex with the lymphoid enhancer factor 1 (LEF1) and T-cell factor 4 (TCF4), upregulating Wnt target genes (*c-myc*, *CYCD1*), responsible for proliferation. Moreover, it increases levels of matrix metalloproteinases (MMPs), thus promoting matrix degradation and tumor invasion. If extracellular Wnt ligands do not bind to FZD and LRP5/6, β-catenin is marked for degradation; a low nuclear pool of β-catenin induces the activity of a transcriptional co-repressor, Groucho, to bind LEF1 and TCF4, repressing Wnt target genes.

The PCP branch of the non-canonical Wnt signaling (Figure 3B) is important for the apical and basolateral polarity of cells, thus, it is a major regulator of the cytoskeletal actin. Along this path, Wnt ligands bind to FZD and a co-receptor, which is either the receptor-like tyrosine kinase (Ryk) or the receptor tyrosine-like orphan receptor (ROR). The ligand binding leads via DVL1 to activation of the disheveled-associated activator of morphogenesis 1 (DAAM1), profilin and the Rho- and RAS-related C3 botulinum toxin substrate 1 (RAC1). DAAM1 activates RhoA, which in turn activates Rho-associated kinase (ROCK), engaged in regulation of the cytoskeleton. RAC1 activates the c-Jun terminal kinases (JNKs), also implicated in regulation of the cytoskeleton, either directly or via the transcription activator protein 1 (AP-1) family. The Wnt/Ca^2+^-dependent non-canonical signaling (Figure 3B) is decisive for the formation of embryonic dorsal axis, cell fate mechanisms, gastrulation, and tissue formation. This path functions without a co-receptor. Upon binding of a Wnt ligand to FZD, DVL1 activates via a G-protein and affects the cGMP-specific phosphodiesterase (PDE) and phospholipase C (PLC) activity, which cleaves the membrane-bound PIP2 into diacylglycerol (DAG) and inositol triphosphate (IP3). IP3 is responsible for the release of Ca^2+^ from the endoplasmic reticulum, elevating intracellular Ca^2+^ pool. The increased Ca^2+^ concentration also leads to activation of Ca^2+^/calmodulin-dependent protein kinase II (CaMKII) and calcineurin. CaMKII regulates NF-κB activity, and calcineurin enhances nuclear factor of activated T cell (NFAT) transcriptional activity, which both regulate transcription of pro-survival genes. Depending on the type of Wnt ligand, the ligand binding can lead to switching of calcineurin function to inhibit β-catenin and the canonical Wnt signaling through activation of the transforming growth factor beta-activated kinase (TAK1) and nemo-like kinase (NLK) (Figure 3B).

There are only a few mechanistic reports on acquired or adaptive resistance of glioblastoma cells or GSCs (glioma stem cells) to RCT (radiation and/or alkylating compounds like TMZ), in the context of the RTK/Wnt receptor signaling pathways. Here, we are focusing on these direct adaptation effects contributing to RCT resistance. We are not taking into account a fast-growing field of Lnc-RNAs, epigenetic regulators (e.g., miRNAs, histone deacetylases), or extracellular vesicles/microenvironment, which have recently been reviewed in detail [139,140,141,142,143,144,145,146,147,148,149,150]. Many papers have shown that, in particular, the alterations in the RTK and Wnt pathways are the hallmark of intrinsic glioblastoma resistance, thus, the modified components of these pathways are putative druggable molecular therapeutic targets [151,152,153,154].

Some 20 years ago, it was shown that the EGFR-mediated activation of the PI3K/AKT pathway was responsible for the resistance to sequential administration of radiation and bi-chloroethyl-nitrosourea (BCNU, carmustine), and was dependent on RAS activity [155]. In another report, it could be observed that acute radiation led to the abundance of the insulin-like growth factor 1 receptor (IGF1R), accompanied by increased secretion of IGF1, which promoted activation of AKT signaling, protecting GSCs from radiation toxicity, thus showing IGF1 receptor signaling is involved in adaptive radioprotection in GSCs [156]. The same group showed that GSCs adapted to the toxicity of repeated irradiation by elevating N-cadherin expression, which resulted in accumulation of β-catenin at the cell surface, thus leading to suppression of the canonical Wnt/β-catenin signaling, reducing neural differentiation, and protecting against apoptosis through clusterin secretion; N-cadherin upregulation was induced by radiation-induced IGF1 secretion, and the radio-resistance could be antagonized by blocking the IGF1R [157].

Further, it was recently published that HGF/c-MET-mediated axis induced a specific β-catenin phosphorylation and Wnt signaling activation, by induction of multidrug resistance-associated protein-1 (MRP-1), leading to epithelial cells stemness-like activation and chemoresistance to TMZ [158]. Another study on TMZ chemoresistance in GSCc found that high mobility group box 1 (HMGB1) upregulated NEAT1, responsible for the Wnt/β-catenin activation. Thus, it was concluded TMZ treatment upregulates HMGB1, which promotes the formation of GSCs via the TLR2/NEAT1/Wnt pathway [159]. Further, it was shown that expression of Wnt/DKK-3/Claudin-5 and TLR4 led to acquired resistance to standard RCT, showing that targeting the Wnt/DKK-3/Claudin-5/TLR4 axis might be a novel therapeutic strategy for GBMs [160]. A brand-new report shows that GSCs secrete extracellular vesicles containing specific molecular cargoes targeting PTEN, which leads to radio-resistance of the recipient cells due to upregulation of the AKT pathway [161] and that the Wnt signaling modulates TMZ resistance in p53-mutant GBM [162]. Further, it was reported that TMZ-resistant U87 cells (despite MGMT deficiency), displayed higher migration capacity and EMT-like phenotype, as well as increased levels of β-catenin, phosphorylated AKT and PRAS40 in the absence of mTOR phosphorylation, indicating the activation of AKT and Wnt/β-catenin paths to be responsible for TMZ chemoresistance [163]. In support of this, it could also be shown that TMZ chemoresistance in glioma cells was acquired by the nuclear accumulation of β-catenin and the upregulation of FoxO3a transcription factor [164], and by the activation of the Wnt/β-catenin signaling involving an ATM/CHK2-independent PI3K/AKT/GSK3β signaling cascade [165]. Furthermore, it was observed that the loss of DOC-2/DAB2 interacting protein (DAB2IP) was responsible for TMZ chemoresistance by inducing the autophagic factor ATG9B, underlying the importance of the activation of the Wnt/β-catenin signaling for acquired resistance to TMZ [166]. Regarding integrin receptor signaling, it could be shown that the adaptor protein and regulator of integrin function, kindlin-3 (FERMT3), regulates glioma cell activity through integrin-mediated Wnt/β-catenin signaling, suggesting FERMT3 activates integrin activity in high-grade gliomas to enhance TMZ chemoresistance [167]. Furthermore, TMZ exposure upregulated PI3 kinase/AKT/NF-κB pathway and reinforced homologous recombination repair of DSBs in glioblastoma cells, the effects that were strongly reduced by siRNA-mediated silencing or pharmacological inhibition of the integrin αVβ3 [168]. It was further shown that expression of histone deacetylase 8 (HDAC8) was responsible for the activation of the Wnt/β-catenin/c-Myc/cyclin D1/Sox2 signaling and glioma cell resistance to TMZ [169]. Moreover, some years ago, it was reported that Wnt/β-catenin pathway regulates gene expression of the MGMT protein, directly leading to TMZ resistance in gliomas [170]. In support of all these findings, it was mechanistically shown, vice versa, that the secreted frizzled-related protein 4 (sFRP4), elicited the non-canonical Wnt/Ca^2+^ pathway, antagonizing the Wnt/β-catenin pathway, and reinforced GSCs differentiation, increasing sensitivity to TMZ [171].

### 3.4. Apoptotic Pathways

**Figure 4:**
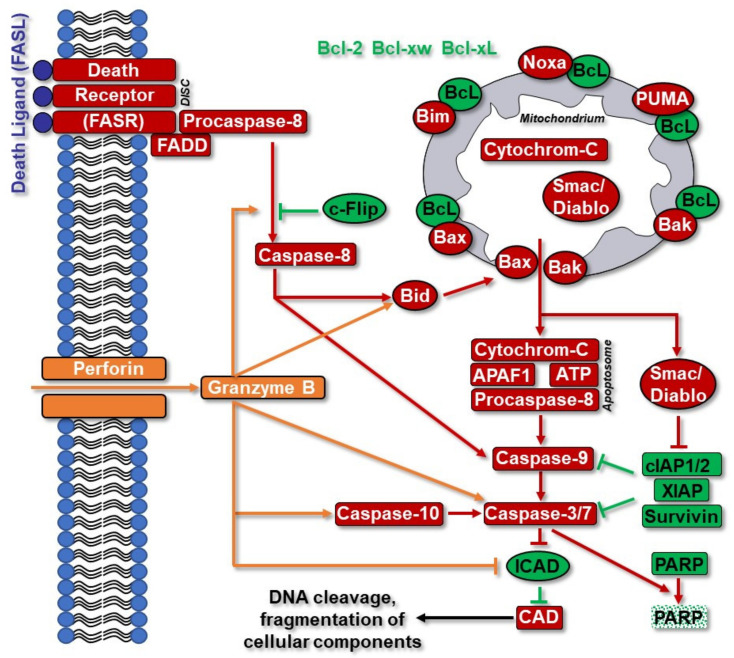
Major apoptotic pathways: extrinsic (death receptor-mediated), intrinsic (mitochondria-mediated) and perforin/granzyme B pathways. For a full description, consult the main text. The green color signals anti-apoptotic, and the red color apoptotic processes.

Apoptosis is a fundamental cell death mechanism that occurs during development and aging as a homeostatic mechanism to control cell populations in tissues, as a defense mechanism in immune reactions and in response to DNA damage. It is a highly complex process that involves an energy-dependent cascade of events and does not elicit an inflammatory response [172] (Figure 4).

This type of programmed cell death exhibits very characteristic morphological and biochemical features and mechanistically occurs through three major pathways: (i) The perforin/granzyme pathway is triggered by host immune system cells, executed via granule-containing perforin and granzymes A and B exocytosis into the immunological synapsis, and incorporation of these molecules by the target cells, where they initiate cell death through caspase-3 activation or by the mitochondrial pathway. (ii) The extrinsic pathway is initiated by the ligand binding to the transmembrane death receptors (DR) from the tumor necrosis factor (TNF) family, such as FASR/CD95, TNF-R, Apo3. Upon ligand binding, cytoplasmic adaptor proteins are recruited and associated with the initiator procaspase-8, forming the death-inducing signaling complex (DISC) and inducing auto-catalytic activation of procaspase-8. Active caspases 8 and 10 directly cleave and activate effector caspases-3 and 7, thus leading to DNA cleavage and fragmentation of cellular components. This pathway can be inhibited by c-FLIP (cellular FLICE (FADD-like IL1β converting enzyme) inhibitory protein), which binds to procaspase-8 and the adaptor FADD (FAS-associating death domain), rendering them ineffective. (iii) The intrinsic pathway is triggered by non-receptor signals that target intracellular elements and culminate in mitochondria permeabilization and release of pro-apoptotic proteins formerly segregated from the cytoplasm. Cytochrome c is one of those proteins that binds the scaffold protein Apaf-1 and procaspase-9, forms the apoptosome, leading to caspase-9 activation. On the other hand, Smac/DIABLO and HtrA2/Omi promote apoptosis by inhibiting IAPs (inhibitors of apoptosis) [173,174,175,176]. The apoptotic signaling pathways in GBMs have recently been described in detail [177].

The perforin/granzyme pathway is not reported to take part in GBMs, probably due to the poor access of immune cells to the brain. Regarding the extrinsic apoptotic pathway, the tumor necrosis factor-related apoptosis inducing ligand (TRAIL) is a potent cell death inducer in GBMs by DR4/5 activation, and the treatment with TRAIL plus TMZ has shown a synergistic survival benefit against GBM in vivo [178]. However, many GBMs show TRAIL resistance, which is often correlated with low procaspase-8 expression and high expression of c-FLIP [179]. FASL is expressed in GBM cell lines and astrocytic brain tumors, and the intracranial delivery of FASL–FADD fusion amplicon concomitant to TMZ treatment enhances survival of mice by inducing apoptosis of tumor cells [180,181]. The expression of FADD has been found to be lower in GBMs compared to normal brain tissues, and its overexpression suppressed proliferation of GBM cells by promoting apoptosis [182]. In respect to the intrinsic pathway, the main induction signal is DNA damage, such as the DSBs induced by RCT, as a protection mechanism against proliferation with an imperfect (damaged) DNA sequence [183].

All three pathways are regulated by p53. TP53 is a major tumor suppressor that integrates diverse stress signals into a series of antiproliferative responses and activates apoptosis by exerting classical transcriptional and less acknowledged non-transcriptional functions [184]. The actual influence of p53 in the response of GBM to RCT is not well-understood. It has been shown that TMZ-induced O^6^mG-triggered apoptosis requires the extrinsic apoptotic pathway in p53wt glioma cells, whereas in p53mt cells this lesion triggers the intrinsic, mitochondrial apoptotic pathway [185]. One possible explanation for the differential sensitivity is the fact that the intrinsic pathway is less effective in inducing apoptosis and requires higher levels of DNA damage [186]. The same group also showed that p53 status can differentially determine the sensitivity of GBM cells to methylating and cloroethylating agents: p53mt cell lines were more resistant to methylating agents (as TMZ), while p53wt were more resistant to cloroethylating agents (as ACNU, BCNU, CCNU) [187]. Mutated p53 GBM cells have also been shown to be more resistant to RT [188]. Similarly, it was demonstrated in another study that p53wt GBMs with a functional p53-mediated DDR could be sensitized to TMZ, whereas p53mt tumors lacking a robust increase in p21 were found resistant. Additionally, tumors with p53mt and weak cell cycle response were found unresponsive [189].

The work of Hirose 2001 can be considered in agreement with the previously cited studies in terms of cell cycle: TMZ-treated U87MG p53wt cells underwent prolonged arrest associated with increased p53 levels, whereas p53-deficient cells underwent a more transient one. However, upon TMZ treatment of p53wt glioma (U87MG) cells neither apoptosis was enhanced, nor survival was reduced [190], a fact corroborated by others [191,192]. In our opinion, those cells started undergoing senescence upon TMZ exposure, which needs longer exposure times, as has recently been published [88,193], which would explain the missing increase in the apoptotic frequency.

Adding complexity to the puzzle, comparing the effect of p53 on response to TMZ in traditional glioma cell lines and brain tumor initiating cells (BTICs), opposite outcomes have been observed: while traditional cell lines with altered p53 expression were significantly more sensitive to TMZ, BTICs with altered p53wt expression/function were more resistant to the drug [194].

The relative contribution of p53 as a predictive/prognostic marker also remains a matter of debate, reflecting the diversity of the in vitro and in vivo findings. While some studies on the analysis of p53 status showed p53-positive patients had a longer survival [195], others did not show any survival increase [196,197,198]. It seems suspicious to assume that such an important cellular player, which coordinates the DDR at so many levels and also appears as a driver gene in gliomagenesis, does not influence the patient’s outcome. A rational analysis of those studies can point out possible explanations for the lack of agreement among them: the stochastic differences due to the relative small size of the samples in each study, the failure to more accurately investigate the p53 functional status (discriminating GOF from other kinds of mutations), the diverse nature of the cohorts (which can present different proportions of GBMs subtypes), and the scarcity of investigation on the regulation of p53 by other regulators, such as non-coding elements.

Another way to infer the importance of p53 in this context is by the manipulation of other elements of the pathway. The use of MDM2 (murine double minute 2) and MDMX (murine double minute X) antagonists, for example, has shown to be an effective way to allow p53 reactivation in p53wt tumors, to induce apoptosis, reduce tumor growth, and to sensitize GBM cells to radiation and TMZ [199,200,201,202,203,204,205,206].

Apoptosis is a direct resultant of the balance between pro and anti-apoptotic forces. IR-induced apoptosis in GBMs is an early event and relies on the G2-arrest. p53mt cells have been found to be deficient in inducing the checkpoints upon IR and turned out to be more resistant [207]. TMZ-induced apoptosis, on the other hand, has been demonstrated to be a late event, occurring more than 48 h after the methylation, and to induce a G2-arrest [88]. p53 controls the expression of the pro-apoptotic and anti-apoptotic Bcl-2 (B-cell lymphoma 2) protein family and of several caspases [208]. Anti-apoptotic proteins Bcl-2 and Bcl-xL have been found to be intrinsically increased, but decreased after TMZ treatment, which was accompanied by activation of caspase-9 and caspase-3. Cytochrome C release and hypophosphorylation of Bad have also occurred, although protein expression of other members of the Bcl-2 family (Bag-1, Bak and Bax) was not altered (Ochs and Kaina 2000). Downregulation of both Bcl-2 and Bcl-xL expression in GBM cells by antisense oligonucleotides led to spontaneous cell death, dependent on executioner caspases 6 and 7 [209,210,211]. The cellular inhibitor of apoptosis protein c-IAP1 and c-IAP2 are critically important proteins that mitigate death signaling from both intrinsic and extrinsic pathways. Chemical inhibition of the anti-apoptotic factors c-IAP1 and c-IAP2 increased TMZ-triggered cell death [212].

Caspases 1, 2, 3, 7, 8, and 9 are constitutively expressed in most human malignant glioma cell lines [213], and many protein targets of active caspases are important markers of apoptosis, indicating a functional caspase activation. Among the substrates of caspases 3 and 7 is PARP, and cleaved PARP (cl-PARP) is a hallmark of apoptosis [214] (Figure 4). In GBM cell lines, IR was found to induce cleavage of PARP starting at day three up to day seven, and partly induced apoptosis [215]. On the other hand, TMZ induced a very mild PARP cleavage, especially at clinically relevant concentrations, indicating TMZ is not efficient in triggering GBM apoptosis [216,217,218,219]. In this respect, the combined treatment was more efficient in provoking apoptosis than either treatment alone, at least in MGMT expressing cells [220].

DNA fragmentation is one of the final phases of apoptosis, carried out by several nucleases. The best characterized nuclease is caspase-activated DNase (CAD), which is tightly regulated by ICAD (inhibitor of CAD) [221]. After apoptosis induction, caspases 3 and 7 and/or granzyme B cleaves ICAD, releasing active CAD that in turn cleaves double-stranded DNA at specific position at the inter-nucleosomal linker [221,222,223]. ICAD has two isoforms, and it has been suggested that ICAD-l, but not ICAD-s, supports the functional production of CAD. In GBM cell lines, the short (s) form is more abundant than the long (l) form [224], but how this correlates with GBM resistance is unclear. Collectively, the evidence points to an important mechanistic role for p53 in apoptosis induction in response to TMZ and radiation, but they poorly induce apoptosis in GBM cells.

### 3.5. Senescence

**Figure 5:**
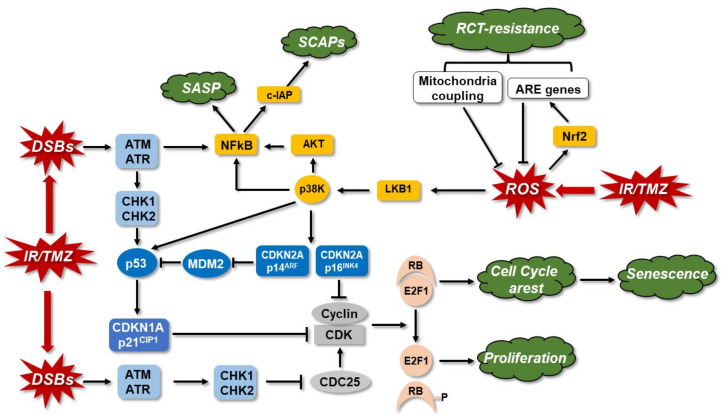
Schematic representation of the pathways leading to cell cycle arrest, senescence, and senescence-associated secretory phenotype (SASP) upon exposure to IR and/or TMZ. For a detailed description, refer to the main text.

Senescence is a highly heterogeneous cellular state characterized by generally irreversible cell cycle arrest, persistent DNA damage, augmented lysosomal activity, resistance to apoptotic stimulus, dysregulated metabolism, and elevated secretion of cytokines, chemokines, and growth factors. Premature senescence, in contrast to replicative senescence, occurs in the absence of telomere erosion and can be induced by a variety of cellular stressors, including IR and chemotherapeutic drugs [225,226]. CDKN1A (cyclin dependent kinase inhibitor 1A, or p21) is a key enforcer of senescence, ensuring the cell cycle blockage by CDK2 inhibition and pRB (retinoblastoma) activation. CDKN2A isoforms p16^INK4A^ and p14^ARF^ also interfere with the cell cycle arrest, by inhibiting the cyclin-dependent kinase 4 (CDK4) and MDM2, respectively [227]. Another critical regulator of senescence is the transcriptional E2 factor (E2F). E2F1 silencing induces replicative senescence markers in cancer cells, while its overexpression turns the cells resistant to senescence [228]. RB, in its dephosphorylated form, binds to E2Fs, forming a repressive complex that inhibits the transcription of the genes necessary for cell cycle progression [88,229] (Figure 5).

For long time, it was believed that cellular senescence was only a tumor suppressor mechanism that permanently arrested cells with accumulated damage and at risk of transformation. However, recent findings are leading to the conclusion that senescent cells can also have deleterious effects on the tissue microenvironment due to the acquisition of a senescence-associated secretory phenotype (SASP), which turns the cells into a pro-inflammatory state with the ability to promote tumor progression [230].

IR and TMZ are strong inducers of senescence in glioma cells and, also, in the subcutaneous, and moreover, in the orthotopic glioblastoma mouse model [88,193]. In vitro, irradiated glioma cell lines die up to 20%, but 60–80% of cells are arrested in the cell cycle, exhibiting senescence markers, such as senescence-associated β-galactosidase (SA-β-gal), H3me9 histone methylation, and p53/p21-positivity as well as upregulating SASP and NF-κB transcriptional activity [231]. Moreover, it has been demonstrated that even RT-induced senescence of non-neoplastic astrocytic cells in the brain promotes tumor growth. In this study, senescent astrocytes showed altered transcriptomic profile with p21 and SASP factors upregulation (e.g., HGF (hepatocyte growth factor), the ligand of MET tyrosine receptor kinase-TRK), which in turn increased MET-driven growth and invasiveness of orthotopically implanted glioma cells. These results surpass the importance of RT-induced senescence in GBM recurrence [232].

TMZ-induced senescence is triggered specifically by the O^6^mG lesion [88]. As mentioned above, upon treatment, p53-proficient glioma cells are arrested in G2/M, upregulating the cell cycle inhibitor p21. The senescence maintenance requires sustained p21 induction. Glioma cells co-treated with TMZ and ATM, ATR, or MRN inhibitors significantly decrease the number of SA-β-gal positive cells, suggesting this DDR axis also plays a role in senescence. TMZ-exposed senescent glioma cells have been shown to exhibit a strong repression of MSH2, MSH6, EXO1, and RAD51, followed by a decrease in MMR and HR activities. As for IR, NF-κB was also shown to be required to induce SASP factors [88].

The enhanced survival capacity of senescent cells and their frequent acquired resistance to genotoxic agents are the result of their intrinsic upregulation of pro-survival, anti-apoptotic mechanisms. One of the relevant mechanisms is referred to as senescent cell anti-apoptotic pathways (SCAPs), in which the Bcl-2 homolog proteins have been shown to play a major role. The inhibition of SCAPs components by senolytic drugs (senolytics) has been recently shown to be an effective mechanism to shift IR/TMZ-induced senescent cells into apoptosis, overcoming resistance [212,233].

### 3.6. Stemness

The cancer cells within a unique tumor are very heterogeneous, and one GBM cell type can have the ability to acquire the phenotype of another. Undifferentiated cells can turn into a more differentiated state, as differentiated cells can transform into a stem-like state. This property is called cellular plasticity, and in gliomas, these tumor stem-like cells (TSCs) (in this review, also GSC, CSC, BTSC (brain tumor stem cell), BTIC, and GIC (glioma initiating cell) are used as equivalent denominations, in fidelity to what is used in the respective cited reference) are thought to be capable of giving rise to cells that express markers of primary neuron and glial cells such as astrocytes and oligodendrocytes, as well as being able to self-renew. However, unlike normal stem cells (NSCs), they function abnormally, and can repopulate the tumor in number and diversity. Beside the ability to generate multiple cell lineages and self-renewal capacity, TSCs must present persistent proliferation and capacity of tumor initiation upon secondary transplantation. Oncogenic signals, particularly from the tumor microenvironment, and their interaction with stem-cell pathways such as Wnt, Notch, or Sonic Hedgehog (SHH), are assumed to be responsible for the induction of TSCs [234,235,236].

In GBM, the specific signaling pathways that characterize GSCs are still under investigation. Patient-derived GBM TSCs usually show strong expression of Nestin, Musashi-1, Sox-2, Oct-4, and BMI1, while differentiated cells express markers according to the cell type and degree of differentiation, such as GFAP (glial fibrillary acidic protein), Tuj-1, NeuN, beta-III-Tubulin, Olig-1. Other common, but not essentially required TSC markers are CD133, CD44, CD15, and L1CAM, among others [236]. Some factors have been shown to be important for TSCs regulation and maintenance of the stemness state. MYC cancer cell survival and proliferation programs play a major role. Beside MYC, chromatin remodeling factors are also necessary: STAT3, SOX2, FOXM1, FOXG1, GLI1, ASCL1, ZFX, NANOG, and ZFHX4 [236].

**Figure 6:**
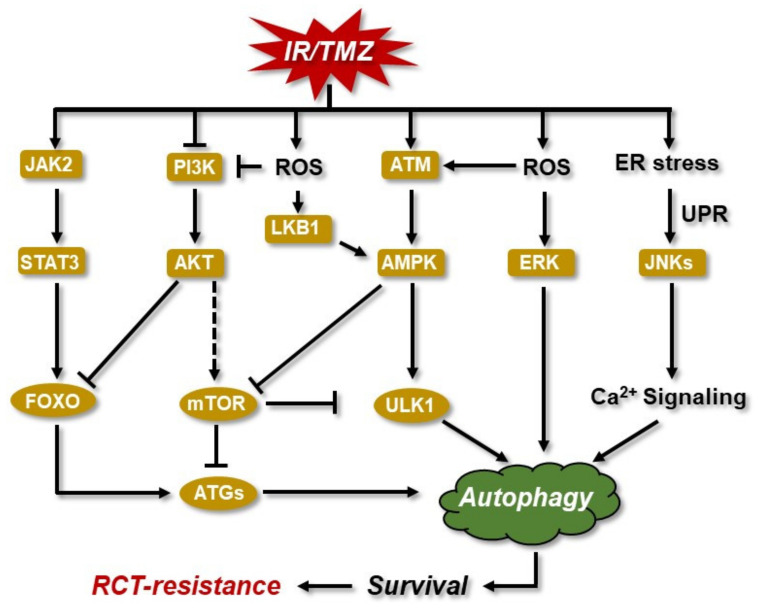
Autophagy regulation and outcomes upon IR/TMZ exposure leading to radiation and chemoresistance. For a detailed description, see the main text.

GSCs are known to be radioresistant, and they exhibit less DNA damage compared to normal glioma cells upon IR. GSCs also respond to the damage with a higher activation of ATM, CHK2, and p53, while the inhibition of pATM (dephosphorylation) significantly increases their rate of apoptosis and sensitization [237,238]. Although with some conflicting results regarding ATM activation upon IR, Lim and colleagues [239] showed an increase in HR activity in GICs, and as for normal glioma cells [94], the knockdown or the pharmacological inhibition of RAD51 rendered the GICs more sensitive to IR [240]. GSC showed a prolonged G2 arrest after irradiation—up to 72 h, leading to delayed FANCD2 foci formation and postponed apoptosis [241,242,243]. IR also enhances the motility, invasiveness, and aggressiveness of GSCs. Motility and invasiveness are the result of activation of the HIF1α axis, which transcriptionally activates the junction-mediating and regulatory protein (JMY), that in turn stimulates GSC migration via its actin nucleation-promoting activity. On the other hand, aggressiveness is attributable to pro-neural-to-mesenchymal transition (PMT), which is associated with the activation of the STAT3 (signal transducer and activator of transcription 3) transcriptional factor. STAT3 is a major signaling hub involved in proliferation, development, and survival [244]. Its downstream targets include FOXO, Bcl-2, Bcl-xL, c-Myc, Survivin, VEGF, and cyclin D, among others, and it is activated upon JAK2 (Janus kinase 2) stimulation by cytokines and growth factors. STAT3 is constitutively activated in malignant gliomas, and its expression corresponds to tumor grade. Apart from its role as a driver of stemness, STAT3 is decisively involved in the induction of autophagy (Figure 6). STAT3 blockage has been shown to inhibit IR-induced malignant progression [245,246].

Curiously, IR can also induce glioma cells de-differentiation, leading them to an acquired GSC-like phenotype. This process is accompanied by the upregulation of the anti-apoptotic protein Survivin, for which downregulation results in the blockade of IR-induced plasticity [247]. IR of the brain microenvironment, beside inducing senescence, also supports glioma stemness and survival via TGM2 (transglutaminase 2) secretion by astrocytes [248], and inhibition of PAF (PCNA-associated factor) turns those cells more sensitive to IR [249].

The treatment of patient-derived GSCs with TMZ revealed in one study that GSC sensitivity also relied on the core MMR proteins (MLH1, PMS2, MSH2, and MSH6), as it is for normal GBM cells. In the same line, TMZ treatment also induced a low frequency of apoptosis and higher senescence in glioma cell lines’ neurospheres [250], and CD133+ CSCs growth was inhibited. Interestingly, TMZ promoted CSCs differentiation, observed as a decrease in the proportion of cells with Nestin marker, while the percentage of GFAP+ (astrocytic lineage), β-tubulin-III+ (neuronal lineage) and GalC^+^ (oligodendroglial lineage) cells increased (Beier et al., 2008). In agreement with that, TMZ treatment of patient-derived xenograft (PDX) mice likewise depleted GSC populations [19]. However, MGMT-negative recurrent tumors were shown to exhibit significantly increased GSCs populations. As it happened for IR, non-stem glioma cells acquired stem-like state after TMZ, and this de-differentiation wasmediated by HIF1α [19]. HIF1α is an upstream transcriptional regulator of *EGF* and *SOX2*, contributing to stemness expression [251]. In another study, newly converted GSCs, when implanted in nude mice, showed a more efficient grafting, fully replenished the tumor population, and displayed a more invasive phenotype, demonstrating the importance of stemness in induced chemoresistance [252].

CD133+ GSCs display a stemness signature associated with SHH signaling, which regulates the expression of stemness genes and is required for sustained glioma growth and survival [253]. Other factors can also interfere with GSCs stemness, self-renewal capacity, and resistance upon TMZ. The transforming growth factor (TGF)-β2 promotes glioma progression and invasion, besides being a primary mediator of glioma-induced immunosuppression [254]. This factor regulates the expression of MMP14 and αVβ3 integrin. In TMZ-treated cells, one study showed MMP14 was translocated to the nucleus, followed by the release of DLL4 (Delta-like canonical Notch ligand 4) protein, a ligand for Notch3 signaling, for which activation caused expression of Nestin and enhancement of the stem-like phenotype; TMZ-treated GSCs showed reduced mRNA and protein levels of TGF-β2, and inferior TGF-β2-mediated invasion capacity [254]. HMG1 are architectural proteins that favor the assembly of multiprotein transcriptional complexes on gene promoters and enhancers. The knockdown of HMG1 sensitized BTSCs to TMZ, reducing their self-renewal and stemness [255].

### 3.7. Autophagy

Autophagy is a conserved catabolic process governed by the PI3K-AKT-mTOR pathway (Figure 6), important for preservation of cellular and organismal homeostasis through balancing sources of energy in response to nutrient stress. Additionally, autophagy provides energetic efficiency by ATP generation and mediates damage control by eliminating non-functional proteins and organelles. Thus, autophagy is thought as a survival mechanism, although its deregulation has been linked to non-apoptotic cell death [256,257]. Macroautophagy (hereby called autophagy), the most relevant autophagic type in cancer, occurs in five steps: the initiation, when the cargos are encompassed by a double-membrane vesicle called autophagosome; the elongation, or extension of this membrane; maturation, when the autophagosome are completely formed; fusion, when the autophagosome fuses with a lysosome, forming the autolysosome, and degradation, when the cargo is degraded by lysosomal hydrolases and the products are recycled back to the cytoplasm [258].

In the context of cancer, autophagy has been shown to play a puzzling role: (i) it can serve as a tumor suppressive mechanism (especially in the initial stages), by restricting proliferation during oncogenic stress; (ii) as a tumor promoter, by providing energy to tumor cells’ survival in poor environments; and (iii) as an RCT-resistance mechanism, by protecting the cells from the induced damage and blocking the apoptotic pathway [257,259,260]. It could be shown that alkylating and chloroalkylating agents such as TMZ and BCNU, respectively, can induce autophagy by inactivation of the PI3K-AKT-mTOR pathway, leading to chemoresistance [261].

Cancers with mutations in RAS or its upstream regulators, as commonly found in GBMs, have higher pro-survival autophagic activity [262,263]. This increase in autophagy occurs through hyperactivation of MAPK and AMPK (adenosine monophosphate-activated protein kinase) signaling. Upon RTK-mediated mitogen stimulation, MAPK drives cellular proliferation/differentiation by MYC and HIF1α activation, switching from catabolic metabolism to anabolic synthesis. Furthermore, MAPK directly regulates AMPK [264,265]. AMPK is an energy sensor that monitors the AMP:ADP:ATP ratio and regulates carbohydrate and lipid metabolism, beside stimulating glucose import and inhibiting mTOR. Several inhibitors or neutralizing antibodies have been developed targeting this pathway and put into clinical trials [266], but no improvement for GBM patients have been observed, so far. One of the explanations for this failure is that blocking hyperactive RAS/RAF/MEK/ERK signaling by MAPK inhibitors in RAS/RAF-mutated cancer cells elevates autophagic flux through relieving LKB1/AMPK/ULK1 (LKB1, liver kinase B1, ULK1, Unc-51-like autophagy-activating kinase) axis and inhibiting glycolysis and mitochondrial functions, which leads to drug tolerance and/or acquired resistance [265]. This path can also be induced by IR/TMZ-induced ROS (Figure 6).

Upon IR, GBM cells have been shown to exhibit an increase in autophagy in a dose-dependent manner, but not apoptosis, as an adaptive response to this insult [267]. The treatment with the autophagy inhibitors 3-methyladenine (3-MA, inhibits the formation of the autophagosome) and bafilomycin A1 (b-A1, inhibits the formation of the autolysosome), radiosensitized the cells, suggesting autophagy acts as a protective mechanism in irradiated glioma cells [267]. In another study, inhibition of autophagy by knockdown of ATG5 reduced radiation-induced apoptosis, and cells were partially protected from cell death after irradiation [268]. One possible explanation for this divergence is that ATG5 is indispensable for autophagic vesicle formation, but it also plays a role in regulating the balance between autophagy and apoptosis. In this context, ATG5 cleavage by Calpain allows the product to translocate to the mitochondria and bind to Bcl-xL, activating caspases without activating autophagy [268]. yH2AX foci, that represent DNA DSBs, were more pronounced and prolonged in cells treated with 3-MA or bA1 and IR in comparison with IR only [267,268,269].

IR also triggers autophagy in GSCs, and CD133+ GSC cells express higher levels of LC3, ATG5, and ATG12, and an increased autophagy frequency compared to the CD133-negative GBM fraction. Autophagy obstruction by inhibition with 3-MA and b-A1 or by ATG5 and beclin1 (BECN1, allows the assembly of the autophagosome from pre-autophagic structures) silencing leads to reduced cellular viability of irradiated cells and decreased neurospheres formation [270]. In normal GBM cells, beside the impairment of autophagy and radiosensitization, the knockdown of BECN1 also resulted in the disruption of nuclear translocation and DNA binding activity of Ku proteins with consequent attenuation of DSB repair, providing evidence of the association of autophagy and repair [271,272]. Again, autophagy is associated with a cytoprotective role in irradiated glioma cells.

DNA-PK is one member of the PI3K family, important in damage sensing, signaling (operating epistatically with ATM) and DSB repair together with Ku proteins. GBM cells intrinsically deficient in DNA-PK are strikingly more sensitive to radiation, compared to the isogenic DNA-PK-proficient GBM cells, and induce autophagy more robustly. In addition, when DNA-PK is inhibited in DNA-PK-proficient GBM cells, autophagy, and sensitization are rescued [273]. The same results are observed in GICs [274]. In this scenario, autophagy induction was not a pro-survival process; it did not have a protective role in GBM cells, in contrast to the results obtained with other autophagy manipulations previously cited above and by others [241,267,275,276,277,278].

mTOR is another member of the PI3K family and integrates both intracellular and extracellular signals. Under nutrient and growth factor signaling, it is activated and inhibits catabolic pathways (such as lysosome biogenesis and autophagy), and its deregulation is associated with many human diseases such as diabetes, neurodegeneration, and cancer [279,280]. The mTOR inhibitor, rapamycin, activates autophagy, but again, it also paradoxically increases radiosensitivity in glioma cells. In GICs, rapamycin decreased tumorigenicity, reduced DNA damage repair, and induced differentiation in other studies. The observed controversial increased radiosensitivity was investigated and proposed to be a result of premature senescence induction [281,282].

Chloroquine (CQ) is a relatively safe drug, widely used against malaria for more than 80 years. It has been investigated as an adjuvant therapy for GBM. Mechanistically, CQ destabilizes lysosomes and activates p53. In gliomas, CQ induces autophagic impairment, ROS formation, mitochondrial membrane potential loss, and apoptosis [283]. When combined with IR, CQ impairs autophagy and the repair of the DNA breaks. On the other hand, it reduces the expression of the anti-apoptotic protein Bcl-2 and increases the effector caspase-3, thus inducing higher levels of apoptosis and radiosensitivity [284].

As IR, TMZ also triggers autophagy, and autophagy markers can be observed in clinical specimens of recurrent tumors [285,286]. However, the autophagic response triggered by TMZ is more complex than the one induced by IR. TMZ-induced autophagy in GBM cells is prevented by MGMT and requires MMR, indicating the O^6^mG lesion is the autophagy-trigger [287]. Different from IR, when autophagy is inhibited in early stages using 3-MA or via BECN1 knockdown, TMZ has its antitumoral effect suppressed (antagonizing with apoptosis), while the inhibition with b-A1 or ATG4C knockdown sensitizes the tumor cells by inducing apoptosis [287,288,289,290]. TMZ-induced senescence requires autophagy, and inhibition of autophagy though 3-MA treatment impairs TMZ-mediated senescence [287,291]. TMZ-induced DSBs directly activate ATM, the upstream kinase of the DDR, which is a component of the ATM/AMPK/ULK1 cascade [289,292] (Figure 6). Inhibition of AMPK, a protein involved in the initiation of the autophagosome formation by interacting with mammalian autophagy-initiating kinase ULK1, augments the cytotoxicity of GBM cells [293]. These results point out to a differential outcome in TMZ response dependent on the stage in which autophagy is inhibited in glioma cells.

Clinically relevant doses of TMZ have been shown to induce autophagy in cells cultured as monolayers and neurospheres, but here, neurospheres were able to maintain a higher basal level of autophagy, suggesting it is associated with the stemness state of the cell. In this regard, the inhibition of autophagy by 3-MA+CQ prevented the generation of TMZ-induced GSC pool [286]. Interestingly, autophagy genes were enriched in less differentiated GBMs (mesenchymal subtype), in which DRAM1 (DNA damage regulated autophagy modulator 1), SQSTM1/p62 (sequestosome 1), and ATG7 were top-ranked in another study [294].

CQ and some of its analogs can also sensitize glioma cells to TMZ, and these observations have pointed to CQ as a new promising adjuvant drug in GBM treatment [216,295,296,297]. CQ and TMZ co-treatment is accompanied by ROS generation, and the application of antioxidants concurrent to CQ suppresses the TMZ chemosensitization effect [216,283]. In this line, the knockdown of NRF2 (NF-Erithroid2-related factor 2), a critical antioxidant transcriptional factor, regulating the antioxidant responsive element (ARE) genes, has also been shown to enhance cell death (see also Figure 5) [298,299]. Finally, the combination (CQ+TMZ) effect has not been observed in p53 mutated cells [289]. Different from the autophagy triggered by TMZ-induced genotoxic stress, the autophagy induced by ROS generation triggered p38 MAPK-dependent degradation of BMI1 protein, belonging to the polycomb group of proteins involved in the epigenetic regulation of nuclear genes, and activated FOXO3 in GICs [297]. FOXO3 activation increased resistance to oxidative stress by enhancing the expression of SOD (superoxide dismutase), catalase and Sestrin, which are the antioxidant enzymes that maintain redox homeostasis [299,300]. In addition, TMZ-induced ROS were shown to activate ERK, leading to protective autophagy and chemoresistance [301].

The large body of evidence suggesting that the induction of autophagy is a chemo-resistance mechanism in GBMs, together with the mechanistic demonstrations of CQ efficiency in overcoming this feature in vitro and in vivo and the long-known safety of this drug have led scientists to include it in many clinical trials. Unfortunately, however, the CQ dose reachable in the tumor has not been enough to achieve autophagy inhibition, and no significant overall survival prolongation has been observed [302].

Finally, GBM tumor progression is also accompanied by a higher endoplasmic reticulum (ER) stress, due to the increased demand for protein folding and transport of newly synthesized proteins [303,304]. The unfolded protein response (UPR) is an adaptive mechanism, also activated during the accumulation of unfolded proteins in the ER. UPR can activate JNKs, which are (via Ca^2+^ signaling) mechanistically linked to autophagy (Figure 6) [305]. The UPR activation is linked to LC3 conversion, which is an essential step for the formation of the autophagosome [306], and ER enlargement is accompanied by the formation of autophagosome-like structures, packed with membrane stacks derived from the UPR-expanded ER [307]. Autophagy then degrades damaged organelles and allows misfolded proteins to be recycled.

Activation of the stemness-reinforcing JAK/STAT signaling has also been involved in induction of autophagy and chemoresistance, thus, the targeting of this pathway in GBM could be therapeutically beneficial [308]. The transcription factor STAT3 is constitutively activated and frequently co-expressed with EGFR in high-grade gliomas [309]. Interestingly, STAT3 inhibition has been shown to overcome TMZ resistance in GBMs by reducing MGMT expression [310].

## 4. Conclusions

In summary, where does the good go?

Although the addition of TMZ to the scheme treatment of GBM improved patients’ overall survival, GBMs have such a mutational background that allows tumor cells to circumvent and adapt to the cytotoxic effects of the RCT and restart growing. It is evident that the benefit of the current therapy is not only very transient, but also interferes with cellular pathways that ultimately turn the recurrences even more aggressive.

Apoptosis is the most desired response in cancer treatment because it is a mechanism of cell death that leaves no inflammation behind. Considering the data available, it is clear that the current treatments induce much less apoptosis than previously assumed. In light of the recent studies in the field, the main response to IR and TMZ in glioma cells is senescence. Senescence has long been considered a beneficial endpoint to be achieved in RCT, due to the permanent arrest in proliferation of the cancerous cells. However, it is now apparent that IR/TMZ-induced senescence also exhibits its adverse effects, particularly concerning induction of the SASP. Whether the IR/TMZ-induced senescent glioma cells in the patient’s tissue can escape senescence and regrow, or whether they create a microenvironment that transforms other resident cells, or both, is a matter of further investigation; but the concrete fact, in this context, is that the induced senescence favors the recurrences.

The new GBM classification, which takes more advantage of the molecular profile of this tumor rather than its mere anatomical or histological classification, is of special significance and leads to numerous past and ongoing clinical trials [311]. It is of great importance to find new, rational ways to interfere together with the standard treatment, taking advantage of these molecular profiles. Some efforts have been made in this scenario e.g., with the use of tyrosine kinase inhibitors, integrin antagonists, and autophagy inhibitors, unfortunately without success in clinical trials. Furthermore, the immunotherapy trials with neutralizing monoclonal antibodies targeting the immune checkpoints, as a promising therapy approach, e.g., in non-small cell lung adenocarcinoma, have remained without any significant therapeutic effect in GBMs.

Despite all the disappointments concerning GBM therapy outcomes, we should remain confident and should not lose hope; many other potential strategies are on the horizon. The use of newly discovered TLS pols inhibitors, or p53 reactivation, are solid fields to be explored, while for example, the use of established senotherapeutics (senolytic and senomorphic drugs) have the theoretical and experimental background necessary to make them good candidates as co-adjuvants to the standard GBM therapy [212,312,313]. To date, there are no clinical trials investigating the potential of these strategies. Additionally, other promising future concepts of personalized and targeted cancer medicine, including those for GBMs, will rely on the sophisticated power of mRNA therapeutics/vaccine-driven cancer immunotherapy [314] and on genome editing and reprogramming by CRISPR/Cas9-based therapeutics [315], although this may still have a long way to go.
